# Age at the time of surgery does not compromise the outcome of deepening trochleoplasty: Results from under and over 30 years old

**DOI:** 10.1002/ksa.12778

**Published:** 2025-07-07

**Authors:** Danko Dan Milinkovic, Felix Zimmermann, Julian Fluegel, Sebastian Schmidt, Peter Balcarek

**Affiliations:** ^1^ Center for Musculoskeletal Surgery Charité‐University Medicine Berlin Germany; ^2^ Arcus Sportklinik Pforzheim Germany; ^3^ Berufsgenossenschaftliche Unfallklinik Ludwigshafen Ludwigshafen am Rhein Germany; ^4^ Department of Orthopeadic and Trauma Surgery University Medical Centre Mannheim, Medical University Mannheim, University of Heidelberg Mannheim Germany; ^5^ Department of Trauma, Hand and Reconstructive Surgery, Departments and Institutes of Surgery Saarland University Homburg Germany

**Keywords:** age, AMADEUS, BPII 2.0, cartilage, patellar instability, PROMs, trochleoplasty

## Abstract

**Purpose:**

This study aimed to evaluate whether age at the time of surgery influences patient‐reported outcome measures (PROMs) in patients undergoing tailored surgical treatment for lateral patellar dislocation (LPD), including deepening trochleoplasty (TP).

**Methods:**

This retrospective cohort study used a prospectively maintained database. The Banff Patella Instability Instrument 2.0 (BPII 2.0) and a numerical analogue scale (0–10) for patellofemoral pain (PFP) and subjective knee joint function were used to assess patients’ disease‐specific quality of life before and after surgery. Preoperative cartilage status was evaluated via the semiquantitative Area Measurement and Depth & Underlying Structures (AMADEUS) scoring system.

**Results:**

Twenty‐nine patients (m/f 4/25) were ≥30 years of age (mean: 35.3; range: 30–51) and formed the study group (SG), and 127 patients (m/f 39/88) were <30 years of age (mean: 20.4; range: 11–29) and formed the control group (CG). The evaluation was performed at a mean of 30 ± 13.2 (SG) and 33.1 ± 13.3 months (CG) post‐operatively (*p* = 0.27). The BPII 2.0 increased from 48.7 ± 21.4 to 85.4 ± 10.7 points (*p* < 0.0001) (SG) and from 44 ± 20.6 to 78.6 ± 18.4 points (*p* < 0.0001) (CG), without any significant difference between the groups at the final follow‐up (*p* = 0.24). PFP and subjective knee joint function also improved significantly in both groups (*p* < 0.0001; *p* < 0.0001), without any significant difference between the groups at the final follow‐up (*p* = 0.08; *p* = 0.3). In the SG and the CG, 88.2% and 89.7% of patients, respectively, achieved an MCID of 10 points calculated for the BPII 2.0 (*p* = 0.99), and no correlation was found for ‘age’ or any evaluated post‐operative PROM (all *p* > 0.1). The AMADEUS scores were 80.1 ± 15 points (CG) and 82.1 ± 14.2 points (SG) (*p* = 0.57), respectively.

**Conclusion:**

Deepening TP, as part of a tailored surgical treatment plan for recurrent LPD, yielded satisfying results, irrespective of patient age at the time of surgery.

**Level of Evidence:**

Level III.

**Abbreviations**
AMADEUS: Area Measurement and Depth & Underlying Structures
BPII 2.0: Banff Patella Instability Instrument 2.0
CD: Caton–Deschamps
DFO: distal femoral osteotomy
IKDC: International Knee Documentation Committee
LPD: lateral patellar dislocation
MCID: minimal clinically important difference
MPFL: medial patellofemoral ligament reconstruction
MRI: magnetic resonance imaging
NAS: numerical analogue scale
PFP: patellofemoral pain
PROM: patient‐reported outcome measure
QOL: quality of life
SG: study group
TP: trochleoplasty
TTO: tibial tubercle osteotomy
TT‐PCL: tibial tuberosity‒posterior cruciate ligament
TT‐TG: tibial tuberosity‒trochlear groove

## INTRODUCTION

Lateral patellar dislocation (LPD) is a common injury in young and active patients, with a high risk of recurrence if left untreated [5, 6, 11]. Several demographic and pathoanatomic factors predisposing patients to LPD have been identified as risk factors for patellofemoral osteoarthritis in the long term [5, 11, 17, 21, 41, 45]. In particular, trochlear dysplasia plays a critical role in patellar malalignment and subsequent cartilage degradation, increasing the risk of osteoarthritis even in younger patients [4, 25, 27, 41].

Surgical management of recurrent LPD aims to restore patellar stability and potentially mitigate further cartilage damage [3, 12, 13]. While various techniques have been established, including medial patellofemoral ligament reconstruction (MPFL‐R) and tibial tubercle osteotomy (TTO), patients with severe trochlear dysplasia often benefit from deepening trochleoplasty (TP) [6, 7, 8, 15, 24, 42]. The influence of patient age on outcomes after patellar stabilization remains debated. TP has traditionally been favoured in younger individuals on the basis of presumed advantages in terms of cartilage remodelling and biological healing capacity; however, robust data supporting age‐related outcome differences are sparse [30, 36, 39, 41]. Recent systematic reviews have reported favourable outcomes across TP techniques, including the Bereiter [20] and Dejour [15] procedures [21, 22, 30, 33]. Nevertheless, given the association between increasing age and progressive cartilage degeneration, it remains unclear whether older patients can achieve outcomes comparable to those of their younger counterparts [2, 13, 15, 23, 34, 39, 40]. Thus, this study aimed to address this shortage by evaluating the outcomes of deepening TP considering patients' age at the time of surgery. It was hypothesized that patients over 30 years of age would achieve patient‐reported outcome measures (PROMs) comparable to those under 30 years of age when undergoing tailored surgical treatment for recurrent LPD, including deepening TP.

## METHODS

This retrospective cohort study used a prospectively maintained database and included 156 patients (male/female: 43/113; age: 22.4 ± 6.8 years) who underwent deepening TP as part of tailored surgical treatment for recurrent patellar instability between 2015 and 2019. Ethical approval for the study was granted by the local ethics committee (Medical Council Baden‐Württemberg F‐2019‐070). To be included, patients had to meet one of the following criteria: a history of at least two LPDs or a first‐time patellar dislocation requiring surgery after at least 6 months of conservative treatment with persistent symptoms. The exclusion criteria included patients with previous MPFL‐R or previous bony procedures such as TTO or distal femoral osteotomy (DFO) and severe degenerative cartilage changes (Iwano Grades III and IV) or generalized osteoarthritis on magnetic resonance imaging (MRI). In addition, patients with patellofemoral pain (PFP) without objective findings of LPD, regardless of their pathoanatomic risk profile, as well as patients with previous ligamentous knee surgery, such as anterior cruciate ligament reconstruction, were excluded.

A standardized clinical and diagnostic work‐up was performed for each patient individually at the time of first presentation. Preoperative MRI and routine radiographs (standing long‐leg axis radiographs and lateral knee radiographs at 30° of flexion) were obtained and assessed for several parameters. These included the degree of trochlear dysplasia, which was classified as absent, low grade (Dejour type A) or high grade (Dejour types B–D). Patellar height was measured via the Caton–Deschamps (CD) index, with values ≥ 1.3 considered elevated. The tibial tuberosity‒trochlear groove (TT‒TG) distance was considered elevated if it was ≥16 mm, and the tibial tuberosity‒posterior cruciate ligament (TT‒PCL) distance was considered elevated if it was ≥25 mm. Valgus malalignment was defined as a mechanical deviation of the hip‒knee‒ankle axis of ≥4° on standing long‐leg radiographs. In cases of clinical suspicion of rotational malalignment (e.g., >70° internal hip rotation in the prone position), torsional MRI was performed, and femoral torsion was assessed according to Jarrett et al. [26], with a threshold of >25° indicating increased torsion. Tibial torsion was assessed according to Strecker et al. [38], with a threshold of 44°, indicating increased tibial torsion.

All patients underwent deepening TP as previously described [9]. The surgical decision for concomitant procedures was based on the individual clinical and anatomical profile and severity of the predisposing risk factors. Deepening TP was considered in patients with Dejour type B or D trochlear dysplasia combined with a positive J‐sign (Grades II–III) and a positive reverse dynamic patellar apprehension test (ReDPAT) ≥ 50° [32], whereas TTO was considered if the TT–TG distance was ≥16 mm, the TT–PCL distance exceeded 24 mm, or the CD index was ≥1.3. Patients with femoral torsion >25° were considered to have derotational DFO, whereas those with valgus deformity ≥4° warranted consideration for varus‐producing DFO [44].

Patient demographic data, including sex, age, height, weight and body mass index, were collected at the initial clinical examination. The preoperative cartilage status was assessed using the “Area Measurement and Depth & Underlying Structures” (AMADEUS) scoring system [28]. The AMADEUS system was applied as a global semiquantitative score, even for multifocal or diffuse lesions, which is consistent with its validated use in the preoperative assessment of overall cartilage status [28]. This scoring system involves three main parameters: the area of the cartilage defect, the morphology and depth of the defect, and the condition of the underlying structures, including adjacent bony defects, subchondral cysts and bone marrow oedema‐like lesions. The cumulative score ranges from 0 (*representing the most severe condition*) to 100 points (*representing no osteochondral defects*) [28].

Pathoanatomic risk factors, as well as patient disease‐specific quality of life (QOL) and PROMs for knee function and pain, were assessed and collected for each patient prior to surgery and at the final follow‐up. The validated Banff Patellofemoral Instability Instrument 2.0 (BPII 2.0) was used to assess patient‐reported disease‐specific QOL, whereas knee joint function and PFP were assessed via a numerical analogue scale (pain/function) with values ranging from 0 to 10, as previously published [42]. All patients who presented with recurrent LPD and fulfilled the radiographic and clinical criteria for deepening TP as a part of tailored surgical treatment were included consecutively, without selection bias. All surgeries were performed by the same senior surgeon.

### Statistical analysis

Continuous data were tested for normality and are presented as the mean ± standard deviation (range). Categorical and dichotomous data are presented as frequency distributions. Differences between groups were evaluated with the Mann‒Whitney *U* test and unpaired *t* tests, and linear regression was used to correlate ‘age’ and ‘AMADEUS’ scores at the time of surgery with post‐operative PROMs. The minimal clinically important difference (MCID) was assessed using the distribution‐based method by calculating half of the standard deviation of the baseline BPII 2.0 score values, as previously reported [14]. All analyses were performed using Prism (version 4; GraphPad Software) with a significance level of 0.05. Post hoc power analysis was performed with G*Power (version 3.1.3).

## RESULTS

**Figure 1:**
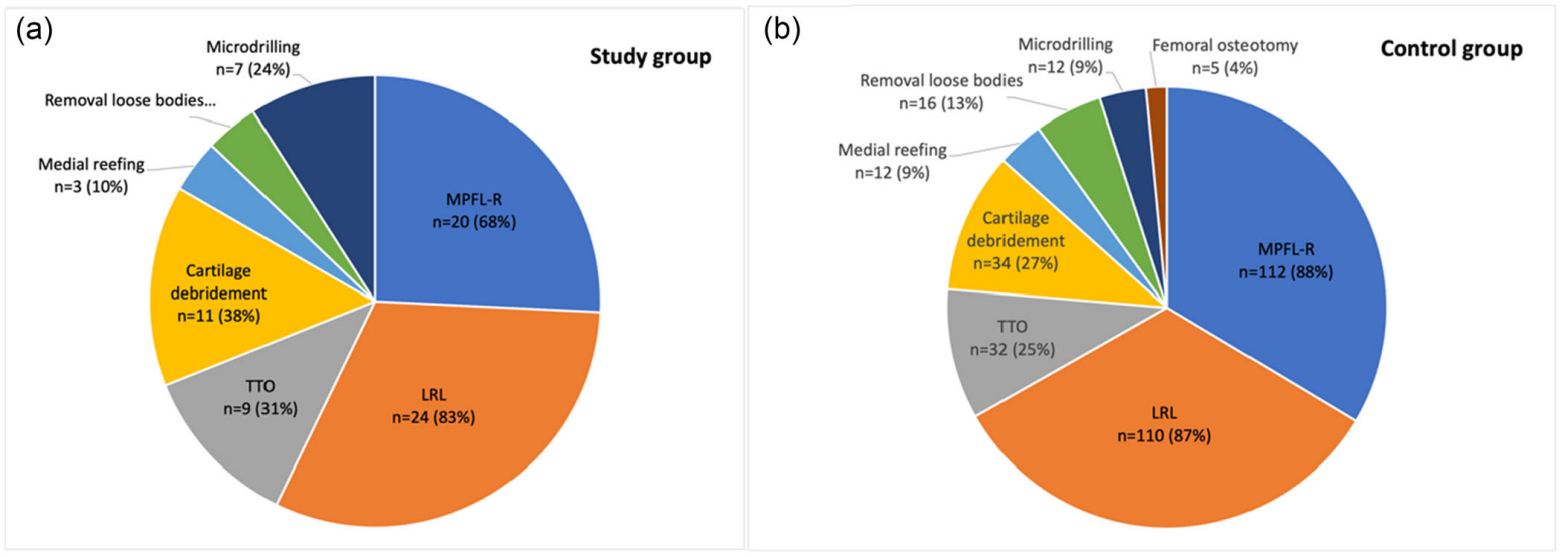
Summary of concomitant procedures in both groups: (a) the study group; (b) the control group. LRL, lateral retinaculum lengthening; MPFL‐R, medial patellofemoral ligament reconstruction; TTO, tibial tuberosity osteotomy.

Twenty‐nine (18.6%) patients (male/female: 4/25) were ≥30 years of age (mean: 35.3 years; range: 30–51) and formed the study group (SG), and 127 (81.4%) patients (male/female: 39/88) were <30 years of age (mean: 20.4 years; range: 11–29) and formed the control group (CG). The evaluation was performed at a mean of 30 ± 13.2 months (SG) and 33.1 ± 13.3 months (CG) post‐operatively (*p* = 0.27). Concomitantly performed procedures are displayed in Figure 1 and did not differ between the groups.

**Figure 2:**
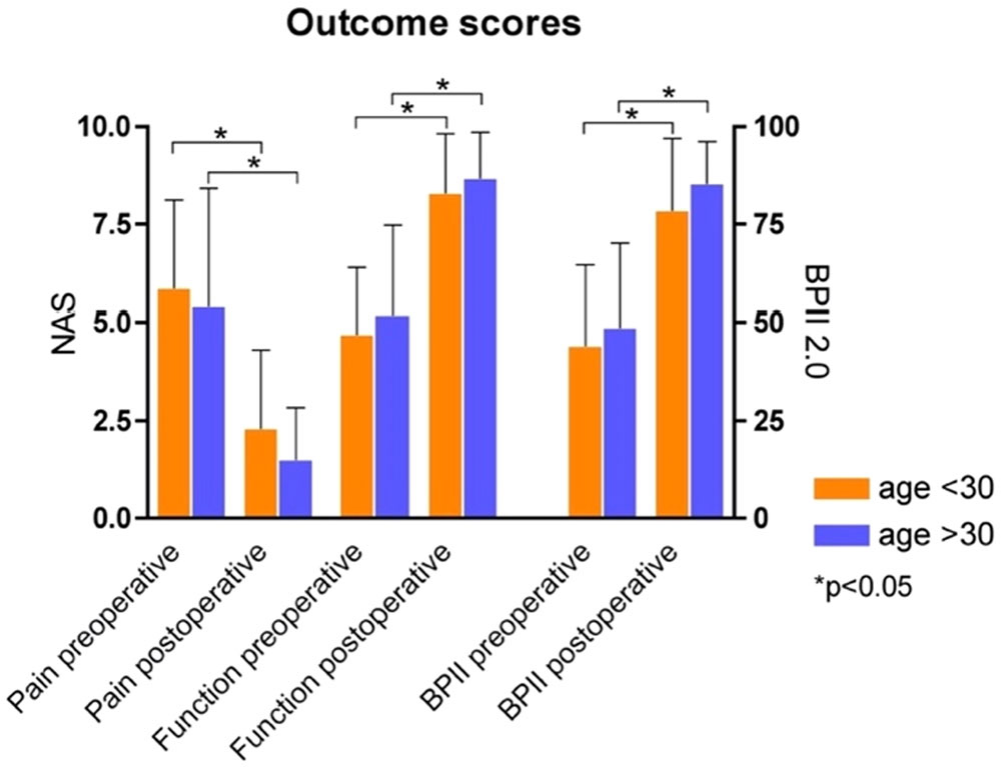
Comparison of preoperative and post‐operative outcome scores in both cohorts. BPII 2.0, Banff Patella Instability Instrument 2.0; NAS, numerical analogue scale.

There was a significant increase in BPII 2.0 values from pre‐ to post‐operative in both the SG and CG (*p* < 0.0001), but there was no significant difference between the groups at the final follow‐up (*p* = 0.24) (power = 0.83; alpha error probability = 0.05 and effect size *d* = 0.6). In addition, PFP and subjective knee joint function improved significantly in both groups (*p* < 0.0001; *p* < 0.0001), again without any significant difference between the groups at the final follow‐up (*p* = 0.08; *p* = 0.3) (Figure 2).

**Figure 3:**
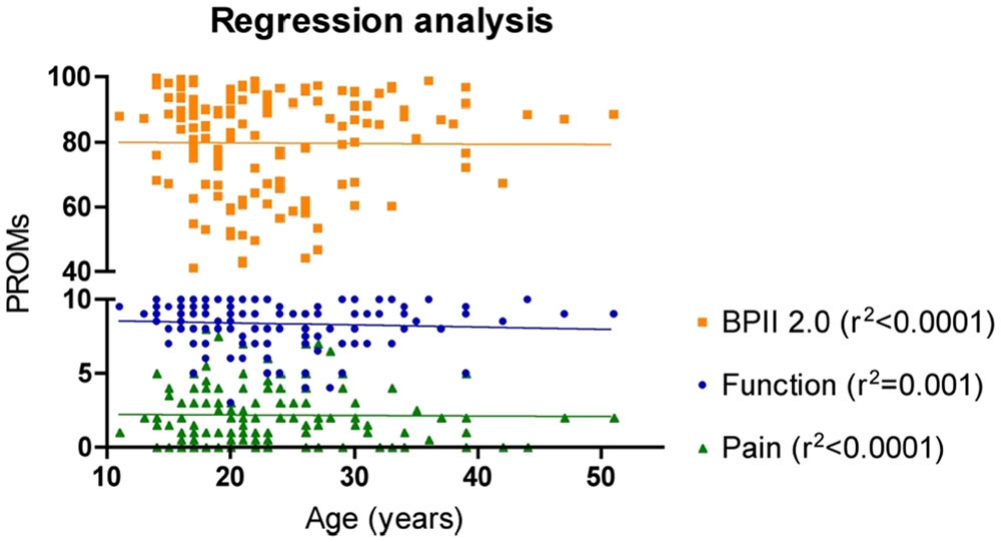
Summary of the correlation analysis for all outcome scores and ages. BPII 2.0, Banff Patella Instability Instrument 2.0; PROMs, patient‐reported outcome measures.

In the SG and the CG, 88.2% (112/127) and 89.7% (26/29) of patients, respectively, achieved an MCID of 10 points, as calculated for the BPII 2.0 (*p* = 0.99). No correlation was found for ‘age’ or any evaluated post‐operative PROMs (all *p* > 0.1; all *r* ^2^ ≤ 0.001) (Figure 3). The preoperative AMADEUS scores were 80.1 ± 15 points (CG) and 82.1 ± 14.2 points (SG) (*p* = 0.57). No correlation was found for the AMADEUS score and post‐operative BPII 2.0 score (*p* = 0.19; *r* ^2^ = 0.01).

## DISCUSSION

The primary finding of this study is that patients aged 30 years and older who underwent deepening TP as part of tailored surgical treatment for recurrent patellar instability achieved improvements in PROMs comparable to those of their younger counterparts. Despite concerns that age‐related factors might influence post‐operative outcomes, no significant differences in post‐operative subjective disease‐specific QOL, pain or function were found between the two cohorts, which shows that age alone should not be a stand‐alone limiting factor in clinical decision‐making when considering deepening TP for the correction of high‐grade dysplasia in patients with recurrent LPD. Notably, despite the expectation of age‐related cartilage degeneration, no significant differences in preoperative AMADEUS scores were observed between age groups, and no associations were found between worse cartilage status and post‐operative PROMs, reinforcing the clinical equivalence of the presented outcomes.

In the patellofemoral joint, recurrent instability is known to predispose patients to traumatic chondral injury and cumulative degeneration of the articular surface over time due to repetitive stress and pathological loading of the cartilage [13, 18, 19, 27, 41]. There are a number of known pathoanatomic factors predisposing to recurrent LPD [1, 5, 7, 8, 9, 25, 35, 45]. Among these, trochlear dysplasia plays a particularly central role, with studies showing that 65%–85% of patients with patellofemoral instability exhibit some form of dysplasia [15, 38]. In addition to its association with instability, trochlear dysplasia has also been identified as a key contributor to patellofemoral cartilage injury and degeneration [4, 6, 7, 25, 29, 44]. Several TP techniques have been introduced, and studies have consistently reported significant functional benefits, low rates of patellar redislocation and high patient satisfaction, which is consistent with the significant improvements in disease‐specific QOL and subjective joint function and pain observed in this study [8, 12, 15, 22, 24, 37, 44]. A recent review by Tan et al. [39], which analysed 21 studies involving 881 knees, compared the results of different TP techniques and concluded that all techniques produced favourable results, with significant improvements in the Kujala, International Knee Documentation Committee (IKDC) and Lysholm scores and low recurrence rates, with no evidence that any particular technique was superior to others. Similarly, Hiemstra et al. [22] evaluated the global outcomes of this procedure in 29 available studies including 998 patients predominantly treated with the thin flap technique (52%) and confirmed that this procedure consistently results in improved clinical and patient‐reported outcomes, with an acceptable complication profile in both short‐ and long‐term follow‐up. In terms of disease‐specific QOL, Orfanos et al. [34] reported significant post‐operative improvements in Kujala and IKDC scores following primary thin‐flap TP combined with MPFL‐R in patients with severe trochlear dysplasia and recurrent instability, whereas Zimmermann et al. [42] reported significant improvements in QOL following tailored surgical treatment in patients with failed primary MPFL‐R, where severe trochlear dysplasia and other predisposing factors were not addressed at index surgeries. There is also significant evidence that the combination of both the MPFL‐R and TP and various other corrective procedures, to address predisposing pathologies, significantly improves clinical outcomes, which is consistent with the findings reported here [8, 10, 31, 39, 42, 43, 44]. Despite the lack of clear age‐specific treatment guidelines and defined thresholds, the general approach has been to avoid this procedure in skeletally immature patients owing to the risk of physeal injury and potential growth‐related complications [12, 31, 36]. The reluctance to perform this technique in older patients, on the other hand, stems from concerns about compromised cartilage vitality and regenerative potential in these patients and the assumption that age‐related factors may affect the success of surgery [12, 39]. However, there appears to be no clear consensus on what the upper age limit should be, as evidenced by the relatively wide age range of patients treated in the available literature, ranging from 8 to 49 years, with a mean of 21.7 years, as previously reported [39]. Considering that age and cartilage status are determinants of outcomes following patellar stabilization procedures, several recent studies have investigated the predictive role of these factors in post‐operative outcomes [19, 21, 25, 45]. Hiemstra et al. [21] investigated age as a factor in post‐operative disease‐specific QOL after MPFL‐R and reported that older patients had lower scores at 12 and 24 months, with each decade of age associated with worse outcomes. In a recent study, age was also outlined as one of the factors that has predictive value for not reaching the MCID in patients who underwent tailored operative treatment for recurrent lateral LPD [14].

Furthermore, Holliday et al. [25] reported that although trochlear dysplasia increased the likelihood of patellar cartilage damage, neither the size nor the severity of these lesions significantly affected QOL scores at 12 and 24 months post‐operatively. Interestingly, although evidence suggests that recurrent LPD is directly correlated with age‐related cartilage deterioration, we observed no significant differences in AMADEUS scores between age groups. Furthermore, no correlation was found between lower AMADEUS scores and reduced post‐operative BPII 2.0 outcomes in this cohort. Nevertheless, prevailing recommendations in the literature suggest that in cases of high‐grade cartilage degeneration, the indication for TP should be considered with caution in light of potentially worse post‐operative outcomes and long‐term joint preservation concerns [16, 30, 37, 40, 43].

## LIMITATIONS

Although this study revealed similar improvements in PROMs across both age groups in patients with very similar risk factor profiles and applied treatment strategies, the results must be interpreted in consideration of several limitations. The retrospective design inherently limits the ability to control for confounding variables and may introduce selection bias. Additionally, this study lacked a clinical correlation with specific outcomes, such as post‐operative patellar apprehension, which could offer a more comprehensive understanding of functional stability. The follow‐up period restricts insight into potential long‐term changes, opening the possibility of further improvement or deterioration in QOL and patellofemoral cartilage over time. Importantly, the absence of post‐operative MRI data is a critical limitation, as it prevents an objective assessment of the effects of deepening TP on cartilage health and the potential development of osteoarthritis. This is particularly relevant in older patients, where cartilage vitality and the risk of degenerative changes are key considerations. Consequently, the long‐term impact of TP on osteoarthritis progression remains unknown, highlighting the need for further longitudinal studies with advanced imaging to better understand these outcomes in all age groups. However, there were no cases of rapid development of osteoarthritis or subsequent revisions in the examined cohorts.

## CONCLUSION

Deepening TP, as part of a tailored surgical treatment plan for recurrent patellar instability, yielded satisfying results, irrespective of patient age at the time of surgery.

## AUTHOR CONTRIBUTIONS

All authors contributed to the study conception and design. Material preparation, data collection and analysis were performed by Danko D. Milinkovic and Peter Balcarek. The first draft of the manuscript was written by Danko D. Milinkovic and all authors commented on previous versions of the manuscript. All authors read and approved the final manuscript.

## CONFLICT OF INTEREST STATEMENT

Danko D. Milinkovic is currently a member of the editorial board and otherwise has no conflicts of interest to disclose. No benefits in any form have been or will be received from any commercial party directly or indirectly related to the subject matter of this article. The remaining authors declare no conflicts of interest.

## ETHICS STATEMENT

This study was approved by the Ethics Committee of Baden‐Württemberg, Germany (F‐2019‐070).

## Data Availability

Raw data can be made available upon request.
